# Automobile Notes

**Published:** 1914-10

**Authors:** 


### AUTOMOBILE NOTES.

---

The Ford announces a reduction in price, August 1, 1914, of about 10% with a further rebate if the next season's sales amount to 300,000—approximately a 50% increase over last year's. It seems to us that it would have been better to install some form of self-starter, electric lights, nonrattling fenders, adequate springs, spark plugs that will not leak and smut, cushions that will not shed tacks and rip apart, curtains that can be easily rolled up, a storage battery, uniform rims, to avoid carrying two extra tires, an oil gage, non-squeaking wind shield and a few other little things. The Ford is an economic, durable car, sound in its essentials and holding up on the road better than many more expensive cars and a good many men would buy it if it cost more.

---

It is claimed that hydrogen peroxid, run slowly through a priming cup while the engine is hot, will remove carbon. Even plain water does the same to some degree by causing minute explosions of steam and because it contains some oxygen in solution. Kerosene, run through in the same way will deliver a satisfying cloud of black smoke but it is questioned by many whether this really represents the removal of encrusted carbon or is due simply to the imperfect combustion of the kerosene itself.

---

It is a bad plan to use all sorts of engine oil. Flat quart cans (tin) inclosed in cigar boxes can be placed in odd spaces under the seat, (as at the end of the gasoline tank) so as to provide for several hundred miles of running.

---

Mailing cases of different sizes, of which every physician has a liberal supply with samples of medicines, are convenient for carrying extra spark plugs, springs, valve caps, polish, kerosene, gasoline for cleaning tubes in mending punctures, trouble lamps, etc.

---

Don't carry tools, metal boxes, etc., in the space about a storage battery. They may cause short circuiting and burning of insulation from wires. We have proved this point by the best and most expensive method—experience. A heavy paper packing box, containing all requisites for mending punctures except gasoline and tools, as well as a flat cigar box containing the trouble lamp and cord, will just about fill the extra space in the battery box.

---

Coffee and sugar sacks are convenient for holding pump, jack, tire tools, etc., overhauls (or overalls if you prefer that derivation), etc.

---

A wooden mallet is convenient for removing and replacing outer tires and is not so liable to damage the rubber.

---

So far as possible, keep all appliances for a given emergency together, using cigar boxes, etc., for assembling small boxes and tools.

---

Remember that the logical principle of a single adequate cause is very liable to be fallacious. For example, in case of a flat tire with leak at the valve, we usually have found that putting in a new plunger does not give relief. There is usually a leak around the valve stem or a puncture also. The same principle is also likely to fail in medical practice.

---

Don't buy an automobile unless you are willing to pay the price. Aside from a cycle car, you can't get away from an expense of about 5 cents a mile as a minimum, distributed about as follows: 1 cent each, per mile for (1) tires, (2) supplies, including chiefly gasoline, engine oil, and grease, (3) repairs, (4) rent, insurance, etc., (5) apportionment of original expense. From this minimum, the cost advances, roughly corresponding to the cost and size of the car, to 30 cents a mile with a chauffeur. Without a chauffeur, you must count labor, either your own or somebody else's, approximately corresponding to half an hour for every hour run.

---

An old long stocking is a convenient holder for inner tubes, tire irons, etc. Tubes, however, should also be packed in a stiff bag, with the valve stems projecting away from each other and packed with paper or cloth to prevent rubbing.

